# Degenerated nucleus pulposus cells derived exosome carrying miR-27a-3p aggravates intervertebral disc degeneration by inducing M1 polarization of macrophages

**DOI:** 10.1186/s12951-023-02075-y

**Published:** 2023-09-04

**Authors:** Xin Zhao, Zhen Sun, Benchi Xu, Wei Duan, Le Chang, Kangwei Lai, Zhengxu Ye

**Affiliations:** 1 Department of Orthopedic, Xijing Hospital, Fourth Military Medical University, Western Changle Road, 710032 Xi’an, Shannxi Provence P. R. China; 2 Xi’an Medical University, 710021 Xi’an, China

**Keywords:** Intervertebral disc degeneration, Nucleus pulposus, Exosome, Macrophages, Inflammatory regulation

## Abstract

**Background:**

Intervertebral disc degeneration (IVDD) is a major contributor to spinal disorders. Previous studies have indicated that the infiltration of immunocytes, specifically macrophages, plays a crucial role in the advancement of IVDD. Exosomes (exo) are believed to play a significant role in intercellular communication. This study aims to investigate the role of exosomes derived from degenerated nucleus pulposus (dNPc) in the process of macrophages M1 polarization.

**Methods:**

Nucleus pulposus (NP) tissue and nucleus pulposus cells (NPc) were collected from patients with intervertebral disc degeneration (IVDD) and idiopathic scoliosis. Immunohistochemistry analysis was performed to determine the number of M1 macrophages in NP tissue. Subsequently, exosomes derived from degenerated NP cells (dNPc-exo) and non-degenerated NP cells (nNPc-exo) were collected and co-cultured with M0 macrophages, which were induced from THP-1 cells. The M1 phenotype was assessed using western blot, flow cytometry, immunofluorescence staining, and qRT-PCR. RNA-sequencing analysis was conducted to examine the expression levels of microRNAs in the dNPc-exo and nNPc-exo groups, and qRT-PCR was performed to investigate the effect pf different microRNA to induce macrophage polarization. Furthermore, western blot and qRT-PCR were employed to demonstrate the regulatory effect of microRNAs carried by dNPc-exo on downstream target signaling pathways in macrophages. Finally, an animal model of IVDD was utilized to investigate the impact of dNPc-exo on inducing M1 polarization of macrophages and its role in the IVDD process.

**Results:**

In this study, we observed an increase in the number of M1 macrophages as the intervertebral disc (IVD) degraded. Additionally, we discovered that dNPc releases exosomes (dNPc-exo) could promote the polarization of macrophages towards the M1 phenotype. Notably, through RNA-sequencing analysis of dNPc-exo and nNPc-exo groups, we identified miR-27a-3p as a highly expressed miRNA in the dNPc-exo group, which significantly influences the induction of M1 polarization of macrophages. And then, we discovered that dNPc-exo has the ability to transport miR-27a-3p and target the PPARγ/NFκB/PI3K/AKT signaling pathway, thereby influencing the M1 polarization of macrophages. We conducted experiments using rat model of IVDD and observed that the exosomes carrying miR-27a-3p actually induced the M1 polarization of macrophages and exacerbated the degradation of IVD.

**Conclusion:**

In conclusion, our findings highlight the significant role of dNPc-exo in IVDD process and provide a basis for further investigation into the mechanism of IVDD and the potential of exosome-based therapy.

**Graphic abstract:**

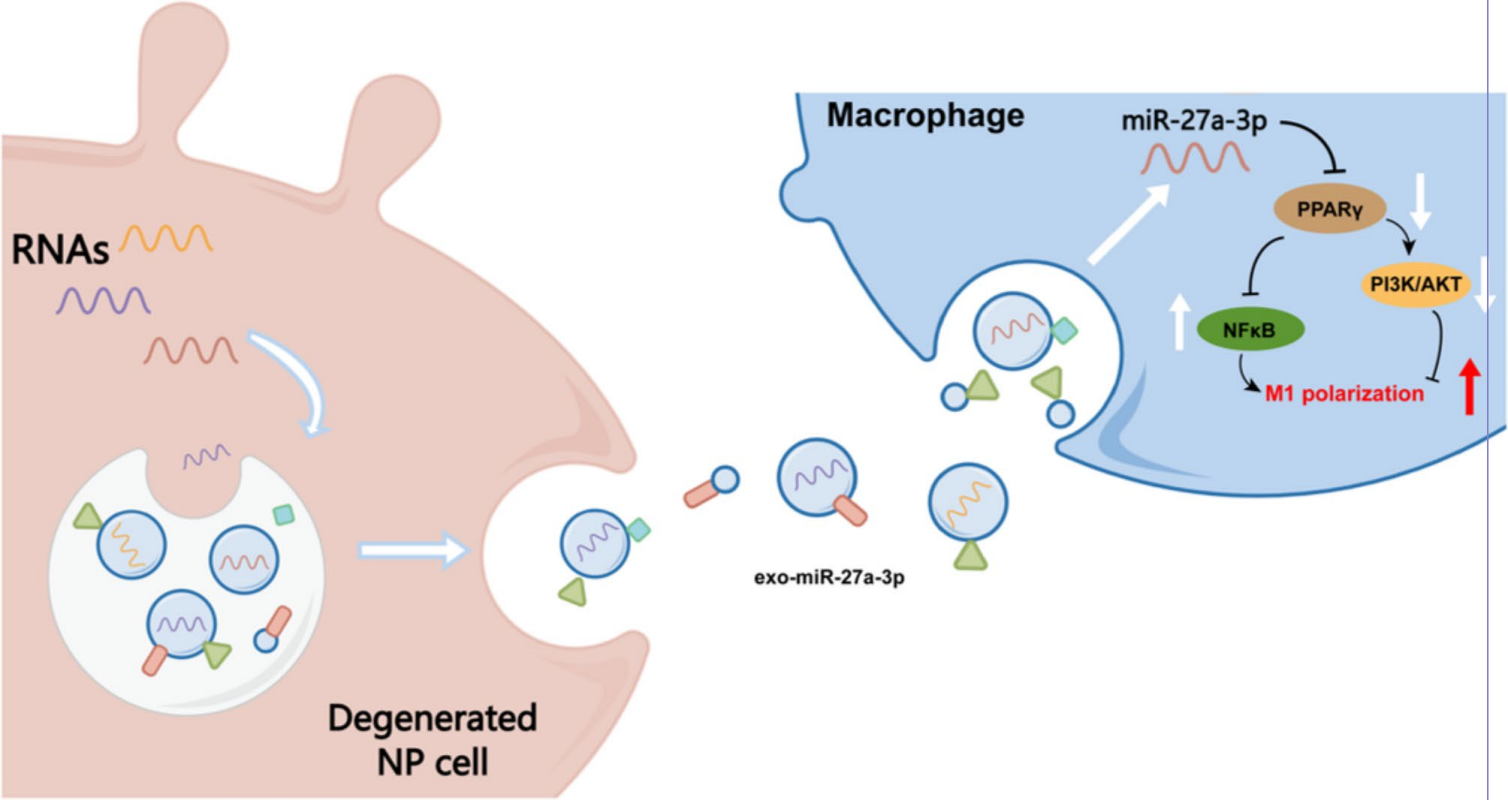

**Supplementary Information:**

The online version contains supplementary material available at 10.1186/s12951-023-02075-y.

## Introduction

The intervertebral disc (IVD) is composed of the adjacent cartilaginous endplates (CEP), the surrounding annulus fibrosus (AF) and the inner nucleus pulposus (NP). Its primary function is to support the stability of the spinal column. However, the accumulation of age, injury, and immune factors can negatively impact the IVD, leading to the IVD degeneration (IVDD) [1–3]. IVDD is the underlying cause of low back pain, which affects over 80% of the global population [4, 5]. Despite ongoing studies, effective therapeutics for the retardation or reversal of IVDD are still lacking. Studies have shown that immunity and inflammation play a significant role in the progression of IVDD, with different subtypes of immune cells contributing to this process. Specifically, macrophages have been defined as a crucial role in the progression of IVDD, with M1 macrophages - also known as ‘classical’ macrophages - being particularly involved in the inflammatory response and release of pro-inflammatory cytokines, which exacerbate the degradation of the IVD [6]. Investigating the unique property of M1 macrophages in the degeneration of IVD could provide a promising theoretical foundation for the therapeutic avenue of IVDD treatment.

Exosomes are extracellular vesicles that secreted by various cells, containing cargoes such as proteins, non-coding RNAs and lipids, with a diameter of 30–150 nm [7]. Studies have demonstrated that exosomes play a crucial role in the interaction and communication between various tissue cells, including IVD cells (CEP cells, AF cells and NP cells), thereby modulating the process of IVDD [8–10]. Feng et al. found that degenerated NP cells (dNPc) can secrete exosomes which promote the degradation of IVD [11]. However, the effect of dNPc-derived exosomes (dNPc-exo) on the M1 polarization of macrophages and the underlying mechanism are still unknown.

This study aimed to investigate the regulatory function of exosomes secreted from dNPc on macrophages and their potential to induce M1 polarization, ultimately intervening in the degradation process of IVD. We found that dNPc-exo carry miR-27a-3p and accelerate the speed of IVD degradation by modulating macrophage polarization. This highlights the significant role of dNPc-exo in the underlying mechanism of IVDD progression.

## Materials and methods

### Sample collection

This study was conducted with the approval of the Ethical Review Committee of Xijing Hospital and written informed consent was obtained from all patients. Disc tissues were collected from 13 patients diagnosed with IVDD and idiopathic scoliosis disease. The patients’ age was 41.9 ± 21.5 (range 16 to 71 years). The NP samples were digested with 0.2% pronase (Gibco, Grand Island, NY, USA) for 40 min with gentle shaking, followed by incubating at 37 °C for 4 h with 0.025% Type II collagenase with gentle shaking. Then, the samples were passed through a 70 mm nylon mesh to remove any remaining tissue pieces. Subsequently, the NP cells (NPc) were incubated at 37 °C in DMEM/F12 medium supplemented with 10% foetal bovine serum (FBS). No ascorbic acid was applied to the media.

### Isolation of dNPc-exo and nNPc-exo

Serial centrifugation with ultracentrifugation was used to isolate dNPc-exo and nNPc-exo. Briefly, non-degenerated and degenerated NPc were inoculated with 1 × 10^6^ cells and grown in basal medium of DMEM-F12. When the cells reached 80% confluence, exosome-free FBS (Thermo Fisher Scientific, Waltham, MA, USA) was used to culture cells. After 48 h, collected the medium and centrifuged at 300×g (4 °C) for 10 min to remove dead cells. Then, obtained the supernatant and centrifuged at 2000×g (4 °C) for 20 min to remove cell debris. Next, the supernatant was centrifuged at 10,000×g (4 °C) for 30 min to remove large EVs. To remove substructures and vesicles larger than 220 nm, we filtered the supernatant through a 0.22-µm filter (Millipore, US). Then, pelleted the exosomes using ultracentrifugation at 100,000×g for 80 min (4 °C). After resuspending in sterile PBS, the exosomes were stored at -80 °C.

### Transmission electron microscopy (TEM)

To determine the exosomes morphology, we dropped 10uL of exosomes suspensions onto a 400 mesh carbon-coated copper grid and allowed it to dry for 20 min. The grids were then rinsed with PBS, fixed with 1% glutamate for 10 min, and rinsed with deionized water. To further stain the grids, they were treated with uranyl oxalate for 10 min.

### Particle concentration and size distribution

The exosomes microstructure was scanned via TEM (Hitachi H-7650, Tokyo, Japan). Nanoparticle tracking analysis (NTA) was used to analyze the exosomes particle concentration and size distribution using ZetaView PMX 110. The samples were diluted with PBS and administered under controlled flow. The data was analyzed via ZetaView Software 8.04.02.

### Protein concentration assay

Pierce BCA Protein Assay Kit (Cat#23,225, Thermo Fisher Scientific, Waltham, USA) was used following the manufacturer’s instructions to determine the exosomes protein concentration. Briefly, exosomes were lysed using RIPA lysis buffer (Beyotime, Shanghai, China) and the proteins were collected. 96-well plate was added with 10 µL protein samples and 200 uL of working reagent. Then, after incubating the plate at 37 °C for 30 min, the absorbance was measured at 562 nm. The protein concentration of exosomes samples was determined using the standard curve method.

### Cell culture and induction

THP-1 human leukemia mononuclear cells were obtained from the Cell Bank of Chinese Academy of Sciences. The cells in exponential growth phase and with good growth conditions were seeded uniformly in 10 cm diameter culture dishes at a 1 × 10^6^ cells/ml density and stimulated with PMA (Sigma-Aldrich, Saint Louis, USA) (100 ng/ml) for 24 h to induce adherent M0 macrophages. The success of the induction was determined by monitoring changes in cell surface markers and morphology.

### Uptake of exosomes in vitro

PKH26 red fluorescence cell linker mini kit (Sigma MINI26-1KT) were labeled with the exosomes following the manufacturer’s instructions. In brief, the exosomes were suspended in Diluent C solution, and PKH26 was diluted in Dilution C. The mixture was incubated at 37 °C for 5 min and then 8 mL of 15% complete medium of exosome-free was added to quench the staining reaction. Then, the exosomes were incubated overnight at 4 °C and centrifuged for 70 min at 100,000×g. Next, PBS (200 µl) were applied to resuspend the exosomes and then incubated with M0 macrophages, which were induced by THP-1 cells, for 24 h. Subsequently, the M0 macrophages were washed with PBS and fixed with 4% paraformaldehyde (PFA). The M0 macrophages were then permeabilized with Triton X-100. After blocking with 10% goat serum, the cells were incubated overnight with the primary antibody against F4/80 (1:100, ab16911, Abcam) and then stained with secondary antibodies and DAPI.

### Detection of Cy3-labelled miR-27a-3p transfer

To investigate whether NPc-exo could carry miR-27a-3p to affect macrophages, NPc was transfected with the Cy3-labelled miR-27a-3p (Tsingke Biotechnology, China). Subsequently, exosomes were purified from the transfected cells and used to co-culture with M0 macrophages. The results were detected using immunofluorescence as described previously. Briefly, F4/80 (1:100, ab16911, Abcam) and DAPI were used to stain M0 macrophages. Olympus FluoView 1200 confocal microscope was used to acquire images.

### Cell transfection

Well-conditioned NPc or THP-1 cells were seeded in cell culture plates. Once the cells reached 60–80% confluency, they were transfected with miR-27a-3p mimic (Ribo Bio, Guangzhou, China), miR-27a-3p inhibitor (Ribo Bio, Guangzhou, China), plasmid of PPARγ (Tsingke Biotechnology, China) or siRNA against PPARγ (#AM16708, Invitrogen by Thermo Fischer Scientific, MA USA), along with the corresponding Negative Control (NC), at a concentration of 100 nM using HiPerFect transfection reagent (Qiagen, Germantown, MD, USA) or Lipofectamine 3,000 (Invitrogen, MA, United States) following the recommended protocols.

### Real-time quantitative polymerase chain reaction (RT-qPCR) analysis

To assess the target genes expression in dNPc-exo, total RNA was extracted using RNAiso for Small RNA (Takara, Shiga, Japan) following the provided protocols. Then, a NanoDrop ND-2000 Spectrophotometer (Thermo Fisher Scientific, Waltham, MA, USA) was used to confirm the purity and concentration of the total RNA. the miRNA 1st strand cDNA synthesis kit (Accurate Biology, China; Cat#AG11717) was used to perform the reverse transcription and all primers were commercially synthesized by Tsingke Biotechnology Co., Ltd. The RT-qPCR was conducted with SYBR Green Premix Pro Taq HS qPCR Kit II (Accurate Biology, China; Cat#AG11719) using the CFX96 Real-Time System (Bio-Rad, Hercules, CA). The primer sequences can be found in Additional Table 1 of this study.

### Western blot analysis

For the identification of exosomes through western blot analysis, Alix, HSP90, and TSG101 markers were used. RIPA lysis buffer (Beyotime, Shanghai, China) was used to collect the exosomes protein samples and Pierce TM BCA Protein Assay Kit (Cat#23,225, Thermo Fisher Scientific, Waltham, USA) was used to determine the concentration. The western blot analysis was performed as previously described. In brief, protein samples were separated on SDS-PAGE gels and detected using appropriate antibodies. Western blot analysis was performed using following antibodies: CD9 (1:1000; #13,174, CST, USA), CD63 (1:1000; ab134045, Abcam), TSG101 (1:1000; #72,312, CST, USA). Anti-mouse IgG or anti-rabbit IgG horseradish peroxidase (HRP)-linked antibody (1: 2000; CST, USA) was used as a secondary antibody, and the chemiluminescent signals were visualized using ECL Western Blot detection kit (WBKLS0100, Millipore).

In the vitro experiment, the protein extracts were separated on SDS-PAGE gels and probed with the following primary antibodies: Anti-CoL2 (1:1000; 28459-1-AP, Proteintech), Anti-iNOS (1: 1000; ab283655, Abcam), Anti-PI3K (1:500; 20584-1-AP, Proteintech), Anti-AKT (1:5000; 60203-2-Ig, Proteintech), Anti-p-AKT (1:5000; 66444-1-Ig, Proteintech), Anti-PPARγ (1: 1000; ab178860, Abcam), Anti-NFκB (1:1000; 10745-1-AP, Proteintech), Anti-MMP3 (1:1000; 17873-1-AP, Proteintech), Anti-CA12 (1:4000; 15180-1-AP, Proteintech), Anti-HIF-1 alpha (1:4000; 20960-1-AP, Proteintech), Anti-TNF alpha (1: 1000; ab6671, Abcam), Anti-GAPDH (1: 1000; ab8245, Abcam). Anti-mouse IgG or anti-rabbit IgG, horseradish peroxidase (HRP)- linked antibody (1: 2000; CST, USA) was used as a secondary antibody, and the immunoreactive bands were visualized by Bio-RAD imaging system (Bio-Rad, CA) and ECL Western Blot detection kit (WBKLS0100, Millipore).

### Flow cytometry analysis

For flow cytometry analysis, single cells were collected and suspended. Cells were then incubated with 1% bovine serum albumin and incubated with CD86-FITC antibody (1:100; 305,213, Biolegend). Subsequently, the cells were determined with a flow cytometer (ACEA NovoCyte) and the data was analyzed using NovoExpress 1.5.6 flow cytometer software.

### Immunofluorescence staining

The cells were fixed with 4% PFA for 30 min. After that, the cells were permeabilized with 0.5% Triton X-100, blocked with goat serum and incubated with primary antibodies against F4/80 (1:50; ab16911, Abcam), iNOS (1:50; ab283655, Abcam). Next, secondary antibodies (1:100; Proteintech) were utilized followed by DAPI staining. The images were required with confocal laser scanning microscope and analyzed via ImageJ software. To facilitate quantitative comparison of immunofluorescence signals, all images within the same experiment were captured using the identical exposure time.

### Exosomes miRNA sequencing

RNA extraction was carried out on dNPc-exo and nNPc-exo samples using RNAiso for Small RNA kit (Takara, Shiga, Japan). RNA quality was assessed for degradation and contamination, and concentration and purity were determined. Small RNAs were reverse transcribed into cDNA to create a cDNA library after being cut into 18-30nt. Illumina HiSeqTM 2500 by Gene Denovo Biotechnology Co (Guangzhou, China) was used to sequence the cDNA. Raw reads were filtered, aligned, and microRNAs were identified. Further analysis included miRNA expression profiles, Principal Component Analysis of miRNA, clustering of miRNA expression patterns, identification of differentially expressed miRNAs (DE miRNA), target gene predictions, and functional enrichment of target genes. DE miRNA was identified based on a fold change greater than 1.5 and a Q-value less than 0.001. Thresholds were established for up-regulated and down-regulated miRNAs.

### Immunohistochemistry analysis

For the change of Col2A1 and Aggrecan content with the IVDD severity, NP tissues were fixed in 4% PFA and 20% Sucrose solution. Next, the tissue samples were sectioned to a thickness of 7 μm and blocked with 5% goat serum. Immunohistochemical staining was performed using Anti-aggrecan (1:400; 13880-1-AP, Proteintech) and Anti-CoL2 (1:400; 28459-1-AP, Proteintech) primary antibodies. The sections were then developed with DAB solution (GeneTech, China) and counterstained with hematoxylin. Microscopy was used to capture images and analyzed using ImageJ software.

### Luciferase reporter analysis

To verify the miR-27a-3p combination capacity with PPARγ, the pmirGLO-luciferase vector was used, into which the wild-type PPARG 3′-UTR and mutant 3′-UTR were inserted. HEK293T cells were co-transfected with 100 ng of reporter plasmid and 30 nM of miR-27a-3p mimic or control using Lipofectamine 3000 transfection reagent and Opti-MEM medium. After 48 h, the dual luciferase reporter assay was performed using the Dual Luciferase Reporter Assay System, and the firefly luciferase activity was normalized using Renilla luciferase activity. The corresponding primers are listed in Supplementary Table.

### IVDD rat model construction and experiment

Animal procedures were followed the guidelines of the Institutional Ethics Review Board of Xijing Hospital. The rat IVDD models were constructed by needle puncturing, as previously described [12].

To investigate the impact of miR-27a-3p on macrophages M1 polarization and IVDD progression in vivo, we extracted NPc from Sprague-Dawley (SD) rats and regulated the expression of miR-27a-3p using mimic or inhibitor. The exosomes of the manipulated NPc were then collected. Then, SD rats (200–250 g) were used to construct IVDD model. In brief, Hypnorm and Dormicum were used to anesthetize the SD rats. The caudal spine was identified by palpation. A longitudinal incision was made in the rat tail skin, and the muscle tissue and subcutaneous connective tissue were separated. Next, an 18-gauge sterile needle was inserted into the disc center to a 5 mm depth, rotated 360 degrees and held for 30 s. Then, a total of 5µL rat-miR-27a-3p-mimic or rat-miR-27a-3p-inhibitor was injected into the rat model via a 27G needle. We monitored the rats daily after surgery to ensure their health condition. After 4 weeks, the rats were put to death and IVDs were obtained from different groups for further experiments. The degenerative control (DC) group consisted of rats treated with PBS after puncture surgery, while the normal control (NC) group consisted of rats that underwent no surgery. A total of 30 SD rats were used for the animal experiment. The primer sequences of miR-27-3p mimic or inhibitor can be found in Additional Table 1.

### X-ray and micro-CT evaluation

The disc height of IVD was assessed using X-ray (GE XR650, USA) Digital X-ray fluoroscopy and micro-computed tomography scanning (PerkinElmer, Waltham, USA). Radiographs of the caudal vertebrae at the experimental level (Co7/8) were taken from rats both before and four weeks after surgery. The disc height index (DHI) was calculated using Image J software and a published method [12], and the DHI% (postoperative DHI/preoperative DHI) was used to express changes in DHI. 3D and 2D reconstructions were generated via CTvox software.

### Magnetic resonance imaging (MRI) evaluation

For the analysis of rat tail data, a MRI scanner (Philips Eclipse, Aachen, Germany) was utilized. The NP structure was evaluated using a T2-weighted sequence. The parameters for the MRI scanner were set as follows: a spin-echo repetition time of 3000ms, an echo time of 90ms, and no phase wrap. Two researchers separated evaluated the signal intensity of rat intervertebral discs on T2-weighted sagittal images and used the Pfirrmann classification to assess the rat IVDD grades. The data were analyzed with Image J.

### Histological evaluation

The paraffin-embedded tissue specimens were cut into 8 mm sections. H & E staining and immunofluorescence were then performed on the specimens. The histological score of IVD degeneration were evaluated via a grading scale as previously described [13]. Two independent observers assessed the degree of IVDD blindly. A normal intervertebral disc has a histological score of 5 points, while a moderately degenerated intervertebral disc has a score of 6–11 points. A severely degenerated intervertebral disc, on the other hand, has a score of 12–14 points.

### Rat-NPc co-culture with M1 macrophages

To investigate the impact of M1 macrophages on the progression of IVDD, we extracted bone marrow-derived macrophages (BMMs) from 8-week old Sprague-Dawley (SD) male rats. BMMs were extracted following a previously described method [14]. Briefly, the SD rats were euthanized, and the femurs were cut at both ends. MEM-α medium (Gibco, Grand Island, NY, USA) was then flushed through the femurs using a syringe to collect the bone marrow. Tissue debris was removed using a 70 μm cell strainer, and the BMMs were cultured in MEM-α medium supplemented with 10% FBS. To induce differentiation of M0 macrophages into M1 macrophages, we stimulated them with 10 ng/mL LPS (Sigma, L2630). Subsequently, the rat-NPc was seeded in 6-well plates (Corning), while the rat M1 macrophages were cultured in transwell inserts (0.4 μm pores; Corning) placed in the same well as rat-NPc. A 1:1 mixture of DF12 and MEM-α medium was added to the 6-well plates.

### Statistical analysis

**Fig. 1:**
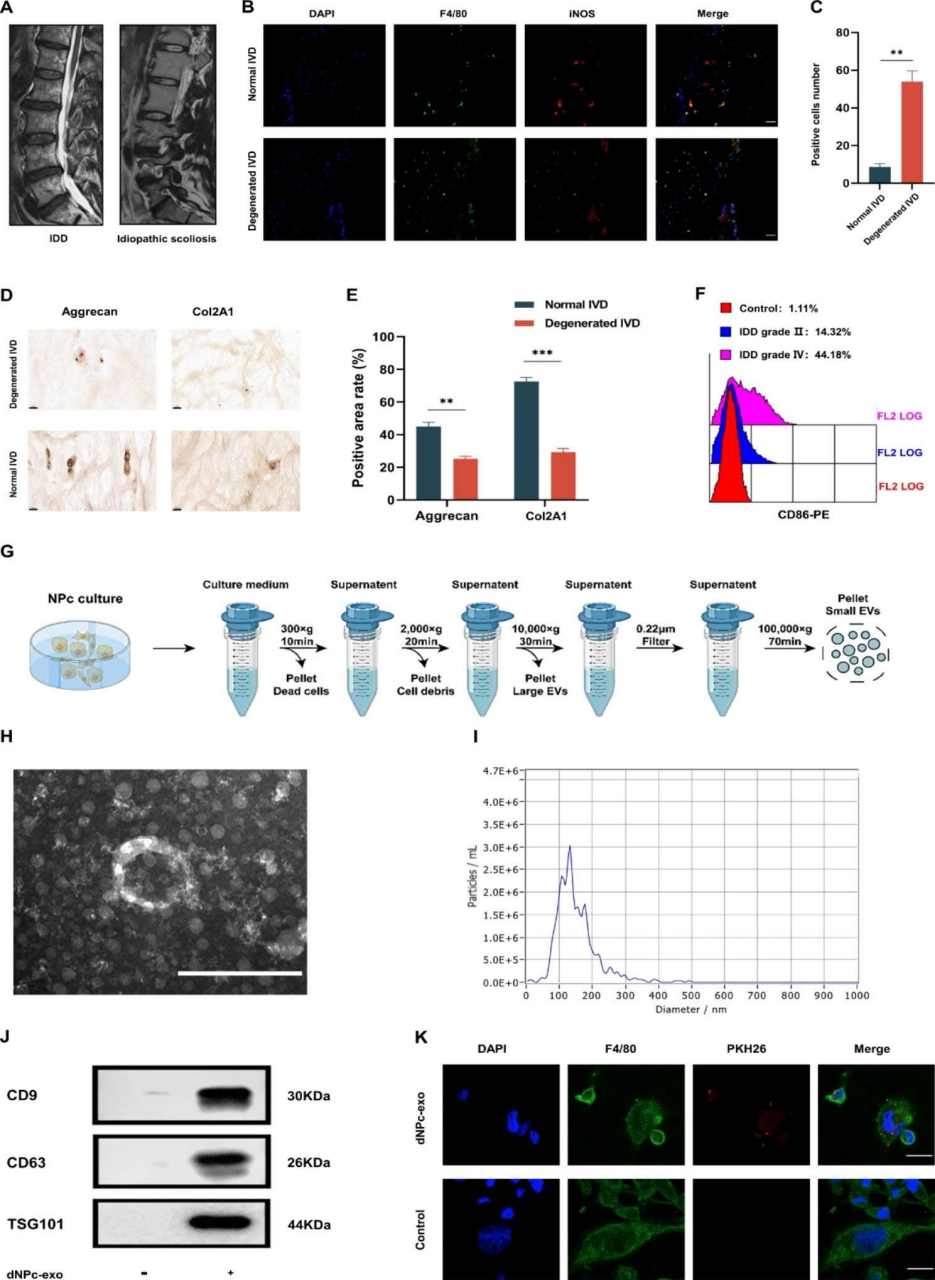
NP cells and NPc-exo isolation and characterization. **(A)** Representative magnetic resonance images (MRI) of idiopathic scoliosis and degenerated IVD. **(B)** Representative confocal images for F4/80 (green) and iNOS (M1 macrophage marker) (red). The nucleus was counterstained with DAPI (blue). **(C)** Quantitative analysis showed the number of iNOS positive cells were significantly increased in IVDD group. **(D)** Expression of Aggrecan and Col2A1 was shown in the two groups by immunohistochemistry. **(E)** Quantitative analysis showed that the expression of Aggrecan and Col2A1 were significantly decreased in IVDD group. **(F)** Representative images of the percentage of CD86 (M1 macrophage marker) positive cells in NP tissues from different IVD grade groups detected by flow cytometry analysis. **(G)** Schematic illustration of NPc-exo isolation using differential centrifugation. **(H)** Typical image of NPc-exo was captured by transmission electron microscopy (TEM). Scale bar = 200 nm. **(I)** Particle size distribution of NPc-exo was examined by nanoparticle trafficking analysis (NTA). **(J)** Western blot analysis of CD9, CD63 and TSG101 markers. **(K)** The M0 macrophages were incubated with PKH26-labeled dNPc-exo and observed. The red fluorescence proved cellular internalization of dNPc-exo into M0 macrophages. Scale bar = 10 μm. The data are expressed as the mean ± SEM. n = 3. *p < 0.05; **p < 0.01; ***p < 0.001; ****p < 0.0001

Statistical analysis was conducted with GraphPad Prism 8.3.0 software. Student’s t-test was used to compare two groups. For group > 2, one-way or two-way ANOVA test was used. Statistical significance was set at P < 0.05. All data were presented as mean ± standard error of mean (SEM). Graphic abstract, Figs. 1G and 6J were created by Figdraw (www.figdraw.com).

## Results

### NP cells and NPc-exo isolation and characterization

In this study, NP cells were acquired from IVDD or idiopathic scoliosis patients (Fig. 1A). The results of immunohistochemistry confirmed the degeneration of IVD by showing a reduced expression of Aggrecan and Col2A1 in degenerated IVD compared to that of the idiopathic scoliosis group (Fig. 1D and E). The number of macrophages, especially M1 macrophages, significantly increased in degenerated IVD tissue, as shown by immunofluorescence staining and flow cytometry analysis (Fig. 1B, C and F, Fig S1). Additionally, western blot analysis was used to detect the NP markers CA12 and HIF-1α (Fig. S3 A, B). Differential centrifugation was used to isolate and collect dNPc-exo and nNPc-exo from NPc culture medium (Fig. 1G). TEM analysis revealed that NPc-exo exhibited a classic “cup-shape” morphology, and the average diameter was around 100 nm (Fig. 1H). The nanoparticle tracking analysis (NTA) found that most exosomes were below 200 nm in size and had a peak diameter of 120 nm (Fig. 1I). Western blot analysis showed high expression of CD9, CD63 and TSG101 in dNPc-exo (Fig. 1J). To confirm if dNPc-exo could be internalized by M0 macrophages, PKH26 was used to label dNPc-exo and added to M0 macrophages cultures. Punctated red fluorescence was observed after incubation, indicating internalization of dNPc-exo by macrophages. F4/80 was used to show the macrophages cellular outline as background staining (Fig. 1K).

### dNPc-exo induced M1 polarization of macrophages via miR-27a-3p

**Fig. 2:**
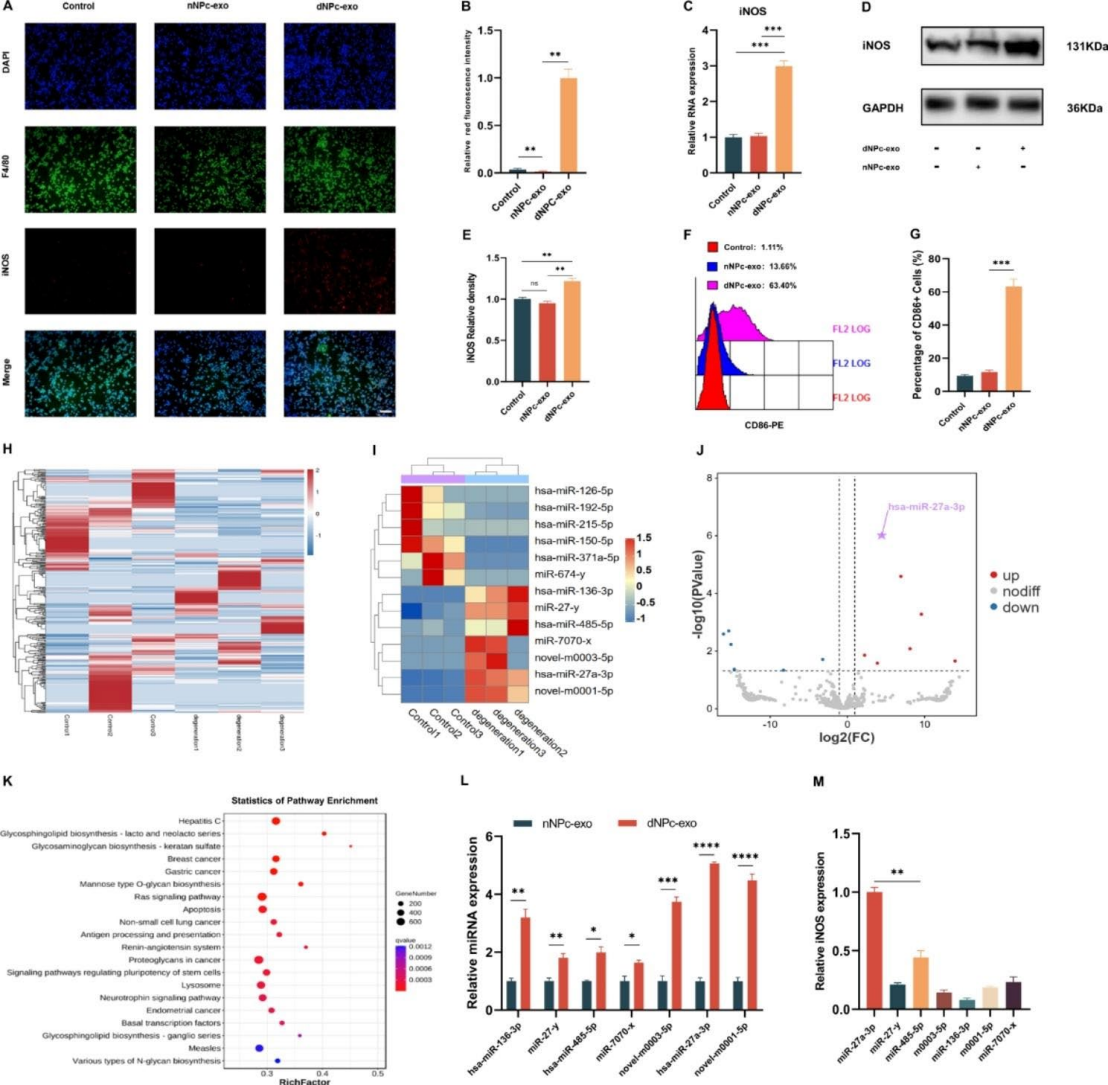
dNPc-exo induces M1 polarization of macrophages via miR-27a-3p. **(A)** Representative images of immunofluorescence staining of F4/80 and iNOS in M0 macrophages after dNPc-exo and nNPc-exo incubation. Scale bar = 100 μm. **(B)** Quantitative analysis of the percentage of iNOS^+^ macrophages in immunofluorescence staining. **(C)** RT-qPCR analysis of iNOS gene expression in macrophages with nNPc-exo and dNPc-exo treatment. **(D)** Western blot analysis of iNOS expression in macrophages with nNPc-exo and dNPc-exo treatment. **(E)** Quantitative analysis of iNOS expression in western blot. **(F)** Flow cytometry analysis of the macrophages. **(G)** Quantitative analysis of the iNOS^+^ macrophages percentage in flow cytometry. **(H)** Heatmap of nNPc-exo group and dNPc-exo group. **(I)** Cluster analysis of RNA-Seq between nNPc-exo group and dNPc-exo group. **(J)** A volcanic map of the differentially expressed genes between nNPc-exo group and dNPc-exo group. **(K)** Pathway enrichment factor map of differential genes (Top20). **(L)** RT-qPCR analysis of seven miRNAs that highly expressed in dNPc-exo group. **(M)** RT-qPCR analysis of iNOS gene expression in M0 macrophages with different miRNAs mimic treatment. The data are expressed as the mean ± SEM. n = 3. *p < 0.05; **p < 0.01; ***p < 0.001; ****p < 0.0001; ns, non-significant difference

To investigate the effect of dNPc-exo on macrophages polarization, dNPc-exo and nNPc-exo were co-cultured with M0 macrophages for 48 h, respectively. Following analysis showed that dNPc-exo could induce M1 polarization of macrophages compared with nNPc-exo (immunofluorescence staining, Fig. 2A, B. RT-qPCR, Fig. 2C. western blot, Fig. 2D, E. flow cytometry. Figure 2 F, G).

To investigate the mechanism of dNPc-exo induced macrophages M1 polarization, RNA-seq was used to analyze the gene expression of nNPc-exo and dNPc-exo. miRNAs that expressed differently between the two groups were detected and further analyzed by the Kyoto Encyclopedia of Genes and Genomes (KEGG) annotation. Among these microRNAs, seven microRNAs (hsa-miR-136-3p, miR-27-y, hsa-miR-485-5p, miR-7070-x, novel-m0003-5p, hsa-miR-27a-3p, novel-m0001-5p) were significantly upregulated in dNPc-exo. As shown in Fig. 2H and I, the heatmap indicates the two-way hierarchical clustering results of miRNAs. The distribution of miRNA in the two groups were displayed using a volcano plot (Fig. 2J). And then, the KEGG Pathway analysis results showed the relative signaling pathways (Fig. 2K). Next, the expression of seven microRNAs (hsa-miR-136-3p, miR-27-y, hsa-miR-485-5p, miR-7070-x, novel-m0003-5p, hsa-miR-27a-3p, novel-m0001-5p) in dNPc-exo were confirmed (Fig. 2L). Subsequently, miRNA mimics were used to upregulate the seven miRNAs expression in M0 macrophages, and iNOS gene expression was validated by RT-qPCR. Among these miRNAs, miR-27a-3p showed significant effect of inducing M1 polarization of macrophages (Fig. 2M).

**Fig. 3:**
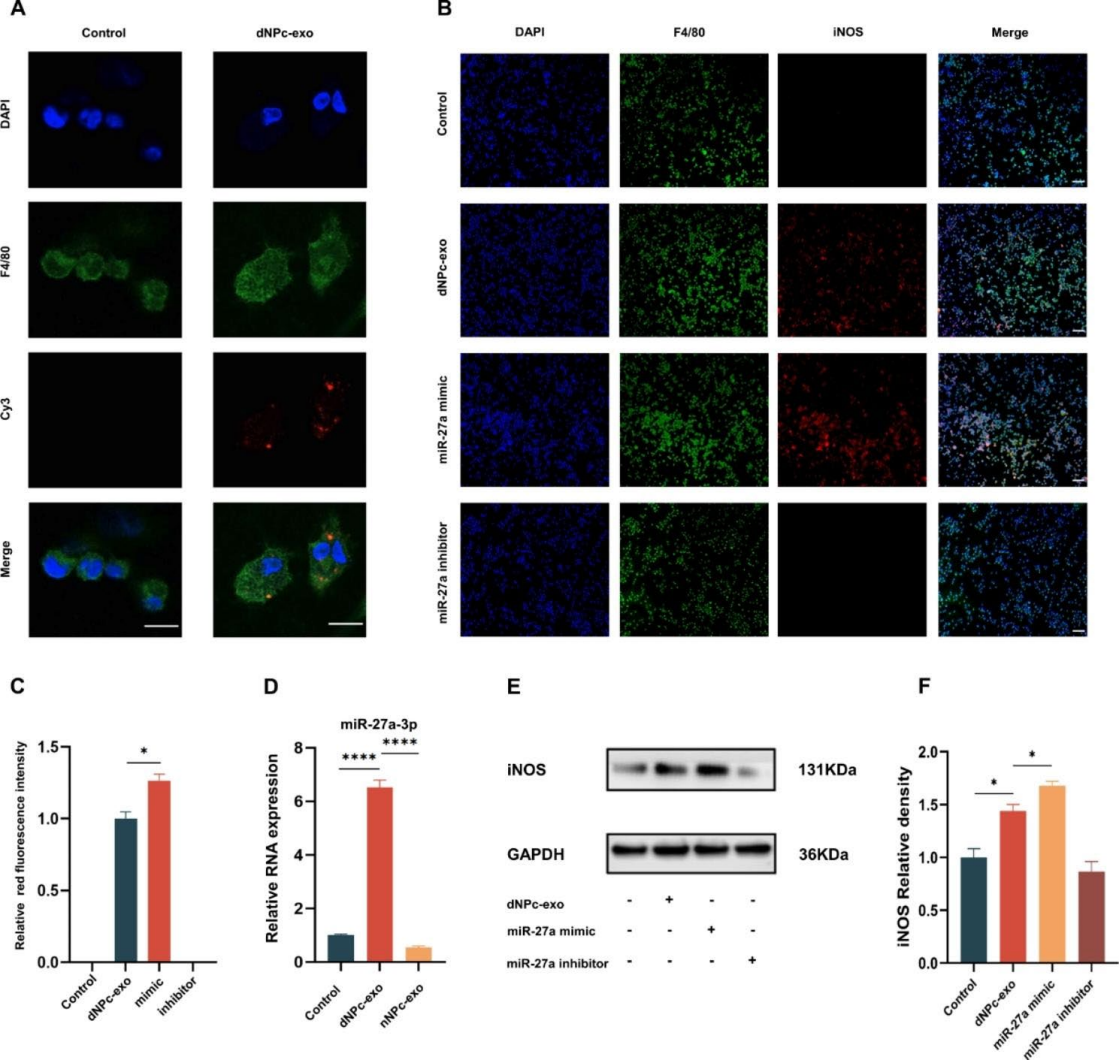
dNPc-exo induces M1 polarization of macrophages via miR-27a-3p. **(A)** Representative images of immunofluorescence staining of Cy3-labelled miR-27a-3p in M0 macrophages. Scale bar = 10 μm. **(B)** Representative images of immunofluorescence staining of macrophages after cultured with dNPc-exo, miR-27a-3p mimic and miR-27a-3p inhibitor (blue, nucleus; green, F4/80; red, iNOS). Scale bar = 100 μm. **(C)** Quantitative analysis of the iNOS^+^ macrophages percentage in macrophages after cultured with dNPc-exo, miR-27a-3p mimic and miR-27a-3p inhibitor. **(D)** RT-qPCR analysis of the miR-27a-3p expression in macrophages of control, nNPc-exo and dNPc-exo group. **(E)** Western blot analysis of iNOS expression in M0 macrophages after treatment of dNPc-exo, miR-27a-3p mimic and miR-27a-3p inhibitor. **(F)** Quantitative analysis of iNOS expression in western blot of M0 macrophages after treatment of dNPc-exo, miR-27a-3p mimic and miR-27a-3p inhibitor. The data are expressed as the mean ± SEM. n = 3. *p < 0.05; **p < 0.01; ***p < 0.001; ****p < 0.0001; ns, non-significant difference

To confirm whether dNPc-exo could transport miR-27a-3p into macrophages, Cy3-labelled miR-27a-3p mimic were incubated with dNPc. The dNPc-exo were isolated and co-cultured with M0 macrophages. Immunofluorescence staining results showed a red fluorescent signal in macrophages co-cultured with the Cy3-labelled dNPc-exo, indicating that miR-27a-3p was transferred from dNPc to macrophages via dNPc-exo (Fig. 3A). After co-culturing, miR-27a-3p expression in M0 macrophages was upregulated in macrophages of the dNPc-exo co-culture group as compared with nNPc-exo and control group (Fig. 3B and C). To confirm the effect of dNPc-exo on macrophages miRNA expression, dNPc-exo was incubated with M0 macrophages. As shown in Fig. 3D, compared with nNPc-exo and control group, miR-27a-3p expression was upregulated in macrophages of the dNPc-exo group. Subsequently, dNPc-exo, miR-27a-3p mimic or miR-27a-3p inhibitor was incubated with M0 macrophages. We found that iNOS expression were up-regulated in macrophages in the dNPc-exo and miR-27a-3p mimic group, indicating that both dNPc-exo and miR-27a-3p could promote M1 polarization of macrophages (Fig. 3E and F). Collectively, these results revealed that dNPc-exo-carried miR-27a-3p could effectively promote M1 polarization of macrophages.

### dNPc-exo-carried miR-27a-3p induced M1 polarization of macrophages via PPARγ/NFκB/PI3K/AKT signaling pathway

**Fig. 4:**
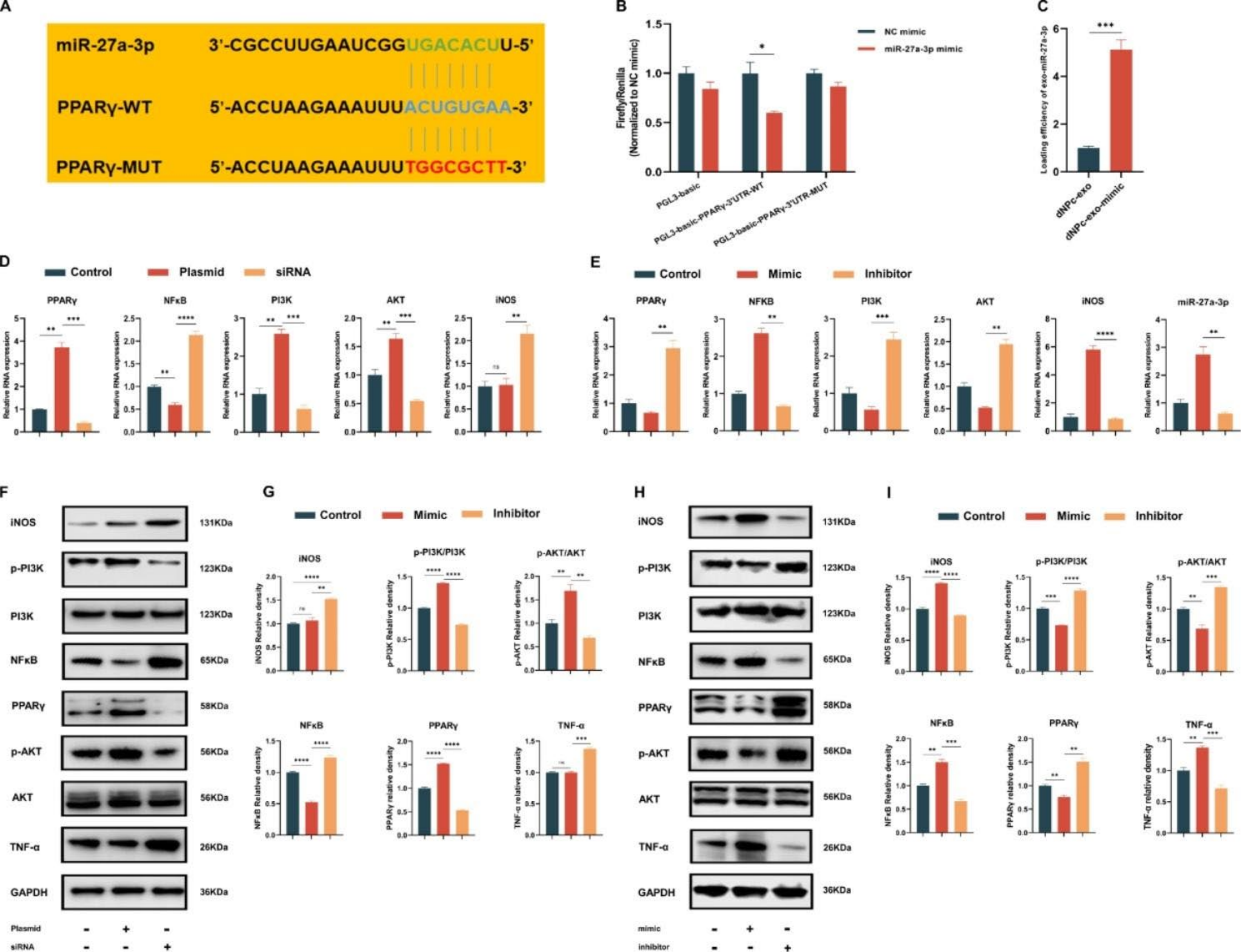
dNPc-exo-carried miR-27a-3p accelerates the M1 polarization of macrophages via regulation of the PPARγ/NFκB/PI3K/AKT pathway. **(A, B)** A bioinformatics analysis predicted the binding sites between miR-27a-3p and PPARγ, confirmed by dual-Luciferase reporter assay. **(C)** The loading efficiency of miR-27a-3p of exosomes. **(D, F, G)** RT-qPCR and western blot analysis of PPARγ, NFκB, PI3K, AKT, iNOS and TNF-α gene expression in M0 macrophages after treatment with PPARγ plasmid and siRNA. **(E, H, I)** RT-qPCR and western blot analysis of PPARγ, NFκB, PI3K, AKT, iNOS, TNF-α and miR-27a-3p gene expression in M0 macrophages after treatment with miR-27a-3p mimic and inhibitor. The data are expressed as the mean ± SEM. n = 3. *p < 0.05; **p < 0.01; ***p < 0.001; ****p < 0.0001; ns, non-significant difference

To determine the pathway targets of miR-27a-3p on macrophages, the potential target genes were predicted via Targetscan (http://www.targetscan.org/). We discovered that PPARγ has a binding sequence with miR-27a-3p (Fig. 4A). This was further supported by the luciferase reporter assay (Fig. 4B). Then, we found that the dNPc-exo-mimic group, where dNPc was co-cultured with miR-27a-3p mimic, showed a significant increase in the expression level of miR-27a-3p compared to the dNPc-exo group. This suggests that the incubation with miR-27a-3p mimic enhanced the loading efficiency of miR-27a-3p into exosomes (Fig. 4C). Studies have shown the essential role of PPARγ/NFκB/PI3K/AKT signaling pathway in the regulation process of macrophages polarization [15, 16]. Therefore, plasmid or siRNA was used to regulate PPARγ gene expression in M0 macrophages. The analysis (Fig. 4D, F and G) revealed that PPARγ upregulation led to a significant increase in PI3K and AKT expression, while PPARγ downregulation resulted in a reduction of these proteins. Additionally, PPARγ upregulation led to a decrease in NFκB expression, while PPARγ downregulation increased its expression. The M1 polarization marker iNOS and pro-inflammatory marker TNF-α was found to be downregulated in the PPARγ upregulation group, whereas iNOS and TNF-α was upregulated in PPARγ downregulation group. And then, miR-27a-3p mimic or inhibitor was used to regulate miR-27a-3p expression in M0 macrophages. As shown in Fig. 4E, H and I, miR-27a-3p levels were significantly increased in miR-27a-3p mimic-treated M0 macrophages, while decreased in miR-27a-3p inhibitor-treated M0 macrophages. The results of western blot and RT-qPCR analysis showed that miR-27a-3p mimic downregulated the expression of PPARγ, PI3K and AKT but enhanced the expression of NFκB in M0 macrophages. Conversely, miR-27a-3p inhibitor enhanced the expression of PPARγ, PI3K, and AKT while downregulated the expression of NFκB in M0 macrophages. Additionally, the expression of M1 polarization marker iNOS and TNF-α was increased in the miR-27a-3p mimic group while decreased in the miR-27a-3p inhibitor group. Collectively, the results revealed that miR-27a-3p carried by dNPc-exo can regulate M1 polarization of macrophages by targeting the PPARγ/NFκB/PI3K/AKT signaling pathway.

### Comprehensive verification of dNPc-exo-carried miR-27a-3p and PPARγ/NFκB/PI3K/AKT regulatory axis

**Fig. 5:**
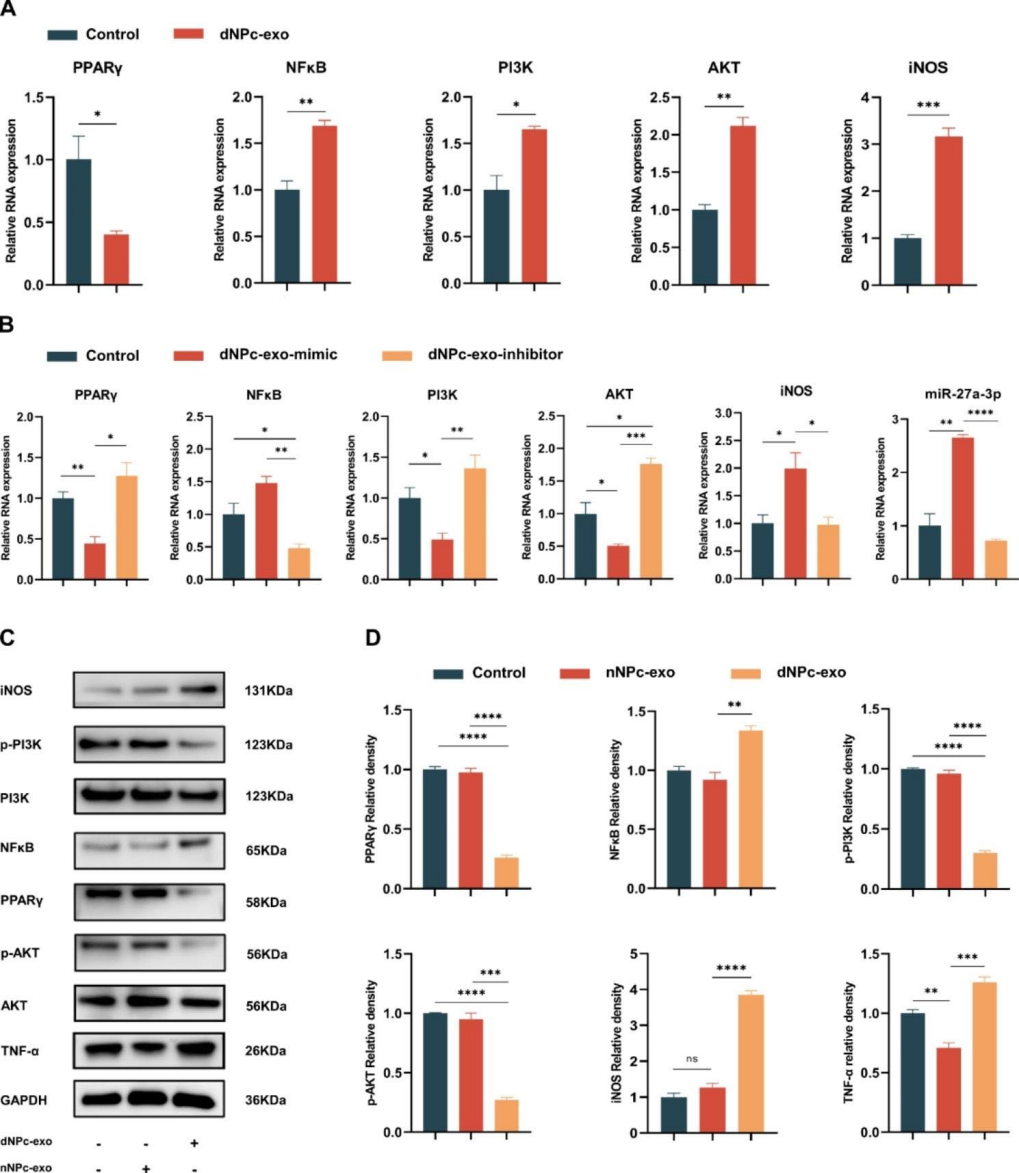
Comprehensive verification of the molecular mechanism of the PPARγ/NFκB/PI3K/AKT regulatory axis of dNPc-exo-carried miR-27a-3p. **(A)** RT-qPCR analysis of PPARγ, NFκB, PI3K, AKT and iNOS gene expression in M0 macrophages after cultured with dNPc-exo. **(B)** RT-qPCR analysis of PPARγ, NFκB, PI3K, AKT, iNOS and miR-27a-3p gene expression in M0 macrophages after cultured with dNPc-exo mimic and inhibitor. **(C)** Western blot analysis of PPARγ, NFκB, PI3K, AKT, iNOS and and TNF-α expression in M0 macrophages after cultured with nNPc-exo and dNPc-exo. **(D)** Quantitative analysis of PPARγ, NFκB, PI3K, AKT, iNOS and TNF-α expression in western blot after cultured with nNPc-exo and dNPc-exo. The data are expressed as the mean ± SEM. n = 3. *p < 0.05; **p < 0.01; ***p < 0.001; ****p < 0.0001; ns, non-significant difference

To investigate the polarization effects of dNPc-exo-carried miR-27a-3p, we collected the dNPc-exo and co-cultured it with M0 macrophages. The western blot and RT-qPCR analysis revealed that the expression of NFκB, M1 polarization marker iNOS and pro-inflammatory marker TNF-α was increased, while the expression of PPARγ, PI3K and AKT was decreased after incubation (Fig. 5A, C and D). Additionally, miR-27a-3p mimic or inhibitor was incubated with dNPc, and the exosomes were collected and co-cultured with M0 macrophages. The results showed that miR-27a-3p could increase the expression of NFκB, M1 polarization marker iNOS, while decrease the expression of PPARγ, PI3K and AKT (Fig. 5B). These findings indicate that dNPc-exo is sufficient to induce M1 polarization of macrophages by transferring miR-27a-3p and targeting PPARγ/NFκB/PI3K/AKT signaling pathway.

### dNPc-exo-carried miR-27a-3p induce M1 polarization of macrophages in IVDD animal model

**Fig. 6:**
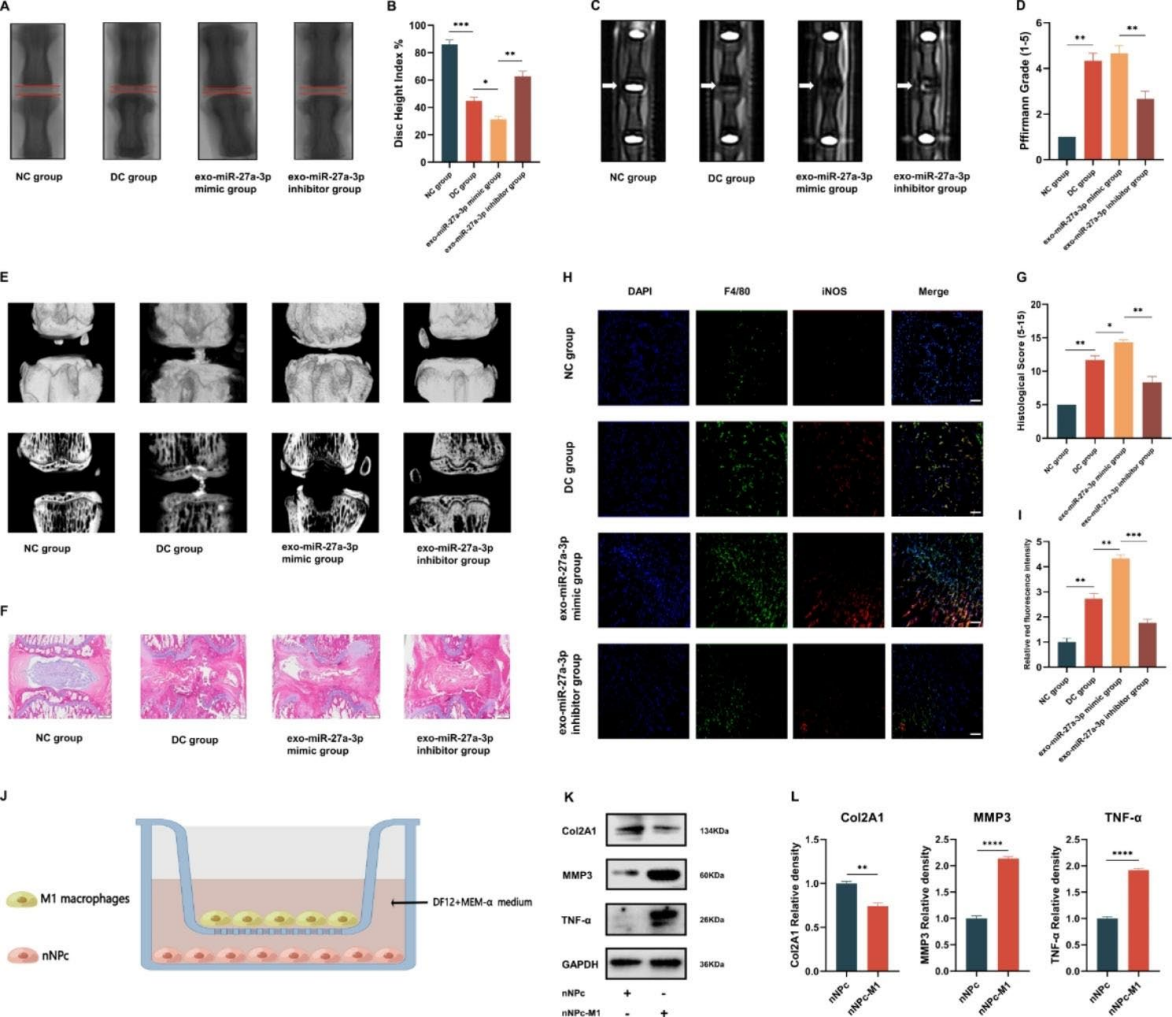
dNPc-exo-carried miR-27a-3p induces M1 polarization of macrophages in IVDD animal model. **(A)** X-ray images of rat spines. **(B)** Quantification of the DHI%. **(C)** MRI images of rat spines. **(D)** Quantification of the Pffirmann grade. **(E)** Micro-CT and 3D reconstruction images of rat spines. **(F)** Representative images of H & E staining of experimental groups. Scale bar = 500 μm. **(G)** The histological scores of different groups after treatment. **(H)** Representative images of immunofluorescence staining of different groups. Scale bar = 100 μm. **(I)** Quantitative analysis of the iNOS^+^ macrophages percentage in immunofluorescence staining. **(J)** Schematic diagram of co-culturing of nNPc and M1 macrophages. **(K)** Western blot analysis of Col2A1, MMP3 and TNF-α expression in nNPc after co-culturing with M1 macrophages. **(L)** Quantitative analysis of Col2A1, MMP3 and TNF-α expression in nNPc after co-culturing with M1 macrophages. The data are expressed as the mean ± SEM. n = 3. *p < 0.05; **p < 0.01; ***p < 0.001; ****p < 0.0001; ns, non-significant difference

To examine the impact of miR-27a-3p on the inducing of macrophages M1 polarization, a rat IVDD model was created using needle puncture. Besides, the rat NPc was isolated and cultured. Subsequently, the rat NPc was co-cultured with miR-27a-3p-mimic, miR-27a-3p-mimic NC, miR-27a-3p-inhibitor, or miR-27a-3p-inhibitor NC. The RT-qPCR analysis revealed that the expression level of miR-27a-3p was upregulated in the miR-27a-3p-mimic group, while it was downregulated in the miR-27a-3p inhibitor group after incubation (Fig. S2). Then, the rat model was intradiscally injected with PBS, exo-miR-27a-3p-mimic, or exo-miR-27a-3p-inhibitor using a 27G needle after surgery. After 4 weeks, the degree of disc degeneration was evaluated using disc height index (DHI) and Pffirmann grade through X-ray, MRI, and Micro-CT examinations. X-ray observations showed minimal changes in DHI of the exo-miR-27a-3p-inhibitor group, which was similar to the NC group (Fig. 6A and B). In the DC group, severe collapse of the IVD space was observed. However, the group treated with exo-miR-27a-3p-mimic showed a more severe collapse compared to the DC group. This indicates that miR-27a-3p could worsen the degradation of IVD.

The results from the MRI images and the Pffirmann grade (Fig. 6C and D) indicate that the IVD of the exo-miR-27a-3p-mimic group was significantly more degenerated than that of the NC and DC group. However, the exo-miR-27a-3p-inhibitor group showed significantly healthier results than the exo-miR-27a-3p-mimic group. Similar trends were observed in the Micro-CT images (Fig. 6E).

To confirm the histology analysis results, H & E staining was performed. The results of the staining (Fig. 6F and G) showed that exo-miR-27a-3p-inhibitor group had a relatively small loss of NP tissue. However, in the DC and exo-miR-27a-3p-mimic group, the NP tissue was gradually replaced by AF. Subsequently, immunofluorescence staining was used to demonstrate the impact of miR-27a-3p on macrophages M1 polarization. The outcome elucidated that the exo-miR-27a-3p-mimic groups had a higher iNOS expression compared to the DC group (Fig. 6H and I). These results collectively suggest that miR-27a-3p can induce M1 polarization of macrophages and contribute to IVDD in vivo.

Then, we isolated the BMMs of rat and used LPS to stimulate it into M1 macrophages. Subsequently, rat-nNPc was co-cultured with M1 macrophages (Fig. 6J), and the western blot analysis revealed a decrease in Col2A1 expression in the nNPc-M1 macrophages group. Conversely, the expression of MMP3 and TNF-α was found to be increased (Fig. 6K and L). These results suggest that M1 macrophages have the ability to enhance the expression of pro-inflammatory cytokines and matrix metalloproteinase in rat-nNPc, thus aggravating the degradation of IVD.

## Discussion

IVD plays a crucial role in maintaining proper spinal function, with the NP being a key component [17]. The healthy NP are immunogenic, it is protected by the AF and CEP [18, 19]. However, with damage and senescence, tiny fissures and tears appear in the AF, exposing the NP to the host immune system and breaking the immune privilege environment [20–22]. Immunocytes infiltrate the IVD and macrophages, the major subset of immunocytes, have been found to significantly contribute to the progression of IVD degeneration through cytokine secretion [23–25]. Specifically, the M1 subtype of macrophages promotes inflammation by releasing pro-inflammatory and inflammatory cytokines, making them a crucial player in IVDD process [26]. Studies have suggested that M1 macrophages might aggravate the progression of IVDD and low back pain [27], but the factors that induce the M1 polarization of macrophages are still unknown. Notably, the extracellular matrix makes up 99% of the structure of NP, with only 1% being cells, which suggests that communication between NPc and other cells may rely heavily on paracrine signaling or indirect cell-cell contact. Study has demonstrated that dNPc could secrete exosomes, which may induce the degradation of nNPc [28]. However, the mechanism of intercellular communication between NPc and macrophages remains unclear.

In this study, we isolated the dNPc-exo and nNPc-exo, and found that dNPc-exo could be internalized by M0 macrophages and induce M1 polarization of macrophages. Next, we demonstrated that dNPc-exo could deliver miR-27a-3p to macrophages and modulate the PPARγ signaling pathway, ultimately affecting the M1 polarization of macrophages. Furthermore, we elucidated that dNPc-exo-carried miR-27a-3p could induce M1 polarization of macrophages and IVD degradation via experiment of IVDD animal model. Our findings suggest that dNPc-exo plays a significant role in IVDD progression by modulating macrophages M1 polarization, and thus accelerating the degradation of IVD.

Studies have shown that exosomes may have a significant role in intercellular communication [29]. These tiny vesicles can encapsulate bioactive molecules such as proteins, lipids, and RNAs from the cells they originate from, potentially influencing the behavior of recipient cells. The exosomes have been shown to regulate the process of IVDD by secreting miRNA [30–32]. Our studies have suggested that AF cells and notochord cells release exosomes. In this study, we further elucidated that dNPc could also release exosomes to affect other cells.

miR-27a-3p has been shown to be involved in cell proliferation, migration and invasion [33, 34]. Yuan et al. discovered that miR-27a-3p has the ability to target RASSF5 and mitigate NPc apoptosis [35]. In our study, we provided evidence that exosome-mediated delivery of miR-27a-3p induces M1 polarization of macrophages. These findings, in conjunction with our current results, indicate that miR-27a-3p plays a crucial role in the advancement of IVDD, and its mechanism of action is complex. Additionally, PPARγ has been shown that plays an important role in downregulating macrophages activation and pro-inflammatory responses [36]. Interestingly, NFκB, PI3K and AKT signaling pathway, which are involved in the regulation of cell proliferation, apoptosis, and protein synthesis, might be the downstream pathways of PPARγ-dependent signaling [37, 38]. Nevertheless, in the field of IVDD, their role still not been further studied.

Here, our study found that dNPc-exo-carried miR-27a-3p can inhibit the PPARγ pathway, which in turn inhibits the downstream PI3K/AKT pathway and accumulates the downstream NFκB pathway. Overall, our results demonstrate that dNPc-exo take part in the intercellular communication and IVD degradation.

This study has several limitations. Firstly, the NPc used for comparison with the degenerated group were acquired from idiopathic scoliosis patients and may not be completely healthy histologically. Therefore, it is possible that the healthy NPc may not have the same characteristics. However, idiopathic scoliosis NP tissue has been widely used as non-degenerated control, and the MRI grading system was used to minimize variations between idiopathic scoliosis NP tissue and healthy NP tissue. Secondly, THP-1 cells were used to stimulate into macrophages. But it would be better to extract macrophages from the blood of volunteers for experiments. Thirdly, the relevant signaling pathway that might combine with dNPc-exo-carried miR-27a-3p was not studied. Further studies are needed to explore the underlying mechanism.

In conclusion, we, for the first time, found that dNPc-exo could induce M1 polarization of macrophages by carrying miR-27a-3p to target the PPARγ/NFκB/PI3K/AKT signaling pathway. This study elucidated that dNPc-exo plays an important role in intercellular communication between NPc and other cells, and aggravates the degree of IVD degradation by inducing M1 polarization of macrophages. This finding reveal the important role of dNPc-exo in IVDD and provide an experimental basis for exploring the mechanism of IVDD and exo-based therapy.

### Electronic supplementary material

Below is the link to the electronic supplementary material.

Supplementary Material 1

Supplementary Material 2

Supplementary Material 3

Supplementary Material 4

## Abbreviations

IVD: Intervertebral disc
CEP: Cartilaginous endplates
AF: Annulus fibrosus
NP: Nucleus pulposus
IVDD: Intervertebral disc degeneration
exo: Exosomes
NPc: Nucleus pulposus cells
dNPc: Degenerated nucleus pulposus cells
nNPc: Normal nucleus pulposus cells
dNPc-exo: Degenerated nucleus pulposus cells derived exosomes
nNPc-exo: Normal nucleus pulposus cells derived exosomes
FBS: Foetal bovine serum
TEM: Transmission electron microscopy
NTA: Nanoparticle tracking analysis
PFA: Paraformaldehyde
SD: Sprague-Dawley
NC: Normal control
DC: Degenerative control
MRI: Magnetic resonance imaging
SEM: Standard error of mean
DHI: Disc height index
