# Supplementary material for: Degenerated nucleus pulposus cells derived exosome carrying miR-27a-3p aggravates intervertebral disc degeneration by inducing M1 polarization of macrophages
Source: J Nanobiotechnology. 2023 Sep 4;21:317. doi: 10.1186/s12951-023-02075-y (PMC10478255; doi:10.1186/s12951-023-02075-y)
Supplement: Supplementary file 1 — Supplementary Material 1 [file 12951_2023_2075_MOESM1_ESM.docx]

Additional Figure **1.**


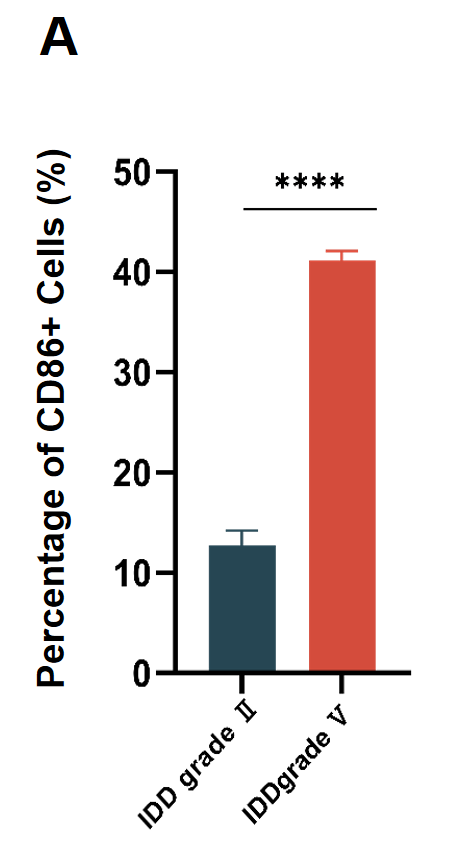


Figure S1. (A) Quantitative analysis of flow cytometry showed that the percentage of CD86 positive cells was significantly increased in IVDD group. The data are expressed as the mean±SEM. n = 3. *p<0.05; **p<0.01; ***p<0.001; ****p<0.0001; ns, non-significant difference.
