# Supplementary material for: Degenerated nucleus pulposus cells derived exosome carrying miR-27a-3p aggravates intervertebral disc degeneration by inducing M1 polarization of macrophages
Source: J Nanobiotechnology. 2023 Sep 4;21:317. doi: 10.1186/s12951-023-02075-y (PMC10478255; doi:10.1186/s12951-023-02075-y)
Supplement: Supplementary file 2 — Supplementary Material 2 [file 12951_2023_2075_MOESM2_ESM.docx]

Additional Figure **2.**


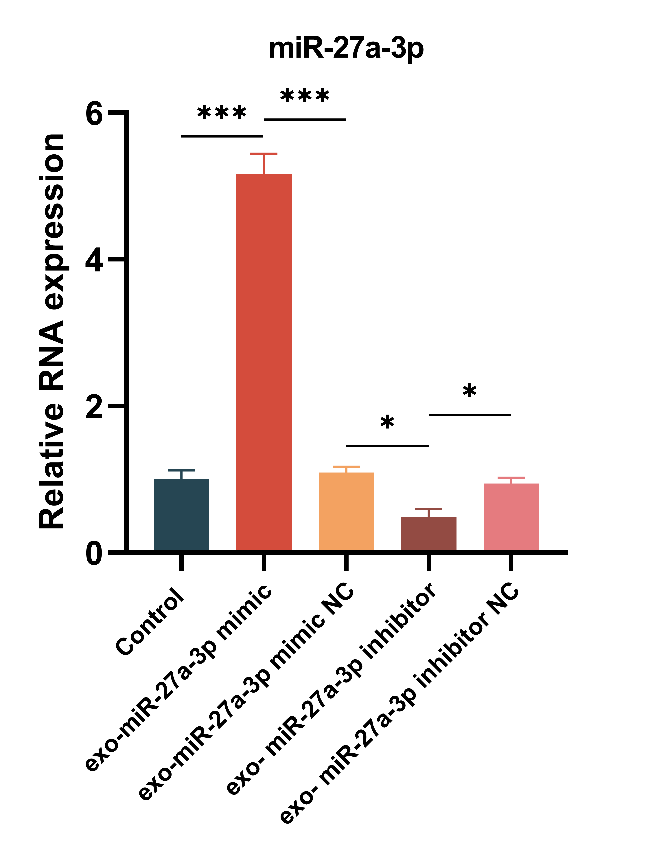


Figure S2. (A) RT-qPCR analysis of the miR-27a-3p expression in exosomes of control, dNPc-exo-miR-27a-3p mimic group, dNPc-exo-miR-27a-3p mimic NC group, dNPc-exo-miR-27a-3p inhibitor group and dNPc-exo-miR-27a-3p inhibitor NC group. The data are expressed as the mean±SEM. n = 3. *p<0.05; **p<0.01; ***p<0.001; ****p<0.0001; ns, non-significant difference.
