# Supplementary material for: Degenerated nucleus pulposus cells derived exosome carrying miR-27a-3p aggravates intervertebral disc degeneration by inducing M1 polarization of macrophages
Source: J Nanobiotechnology. 2023 Sep 4;21:317. doi: 10.1186/s12951-023-02075-y (PMC10478255; doi:10.1186/s12951-023-02075-y)
Supplement: Supplementary file 4 — Supplementary Material 4 [file 12951_2023_2075_MOESM4_ESM.docx]

Additional file

Additional Table **1. The list of primers.**

| Gene | Forward primer | Reverse primer |
| --- | --- | --- |
| hsa iNOS | TTCAGTATCACAACCTCAGCAAG | TGGACCTGCAAGTTAAAATCCC |
| hsa PPARγ | GGGATCAGCTCCGTGGATCT | TGCACTTTGGTACTCTTGAAGTT |
| hsa NFκB | AACAGAGAGGATTTCGTTTCCG | TTTGACCTGAGGGTAAGACTTCT |
| hsa AKT | AGCGACGTGGCTATTGTGAAG | GCCATCATTCTTGAGGAGGAAGT |
| hsa PI3K | CCACGACCATCATCAGGTGAA | CCTCACGGAGGCATTCTAAAGT |
| hsa GAPDH | GGAGCGAGATCCCTCCAAAAT | GGCTGTTGTCATACTTCTCATGG |
| hsa miR-27a-3p | TTCACAGTGGCTAAGTTCCGC |  |
| hsa U6 | CTCGCTTCGGCAGCACA |  |
| Luciferase primer | | |
| hsa-miR-27a-3p mimic | UUCACAGUGGCUAAGUUCCGC | GCGGAACUUAGCCACUGUGAA |
| hsa-miR-27a-3p  mimic NC | UCACACUCCUAGAAAGAGUAGA | UCUACUCUCUGUAGGAGUGGA |
| miR-mimic/inhibitor | | |
| hsa-miR-27a-3p mimic | UUCACAGUGGCUAAGUUCCGC | GCGGAACTTAGCCACTGTGAA |
| hsa-miR-27a-3p  mimic NC | UCACACUCCUAGAAAGAGUAGA | UCUACUCUCUGUAGGAGUGGA |
| hsa-miR-27a-3p inhibitor | GCGGAACUUAGCCACUGAA |  |
| hsa-miR-27a-3p inhibitor NC | UCUACUCUUUCUAGGAGGUGUGA |  |
